# Tetrahydrocurcumin Ameliorates Skin Inflammation by Modulating Autophagy in High-Fat Diet-Induced Obese Mice

**DOI:** 10.1155/2021/6621027

**Published:** 2021-06-14

**Authors:** Jung Eun Kim, Hye Ran Kim, Jin Cheol Kim, Eun Soo Lee, Choon Hee Chung, Eun Young Lee, Bo Young Chung

**Affiliations:** ^1^Department of Dermatology, Soonchunhyang University Cheonan Hospital, Soonchunhyang University College of Medicine, Cheonan 31151, Republic of Korea; ^2^Department of Dermatology, Hallym University Kangnam Sacred Heart Hospital, Hallym University College of Medicine, Seoul 07441, Republic of Korea; ^3^Institution of Genetic Cohort, Yonsei University Wonju College of Medicine, Wonju 26426, Republic of Korea; ^4^Department of Internal Medicine, Yonsei University Wonju College of Medicine, Wonju 26426, Republic of Korea; ^5^Department of Internal Medicine, Soonchunhyang University Cheonan Hospital, Soonchunhyang University College of Medicine, Cheonan 31151, Republic of Korea

## Abstract

Obesity can induce chronic low-grade inflammation via oxidative stress. Tetrahydrocurcumin (THC) is a major curcumin metabolite with anti-inflammatory and antioxidant effects, but little is known about its effects on the skin of obese individuals. Thus, the aim of this study was to investigate the effects of THC on inflammatory cytokine production, oxidative stress, and autophagy in the skin of mice with high-fat diet- (HFD-) induced obesity. Eight-week-old C57BL/6J mice were fed a regular diet, HFD (60% of total calories from fat), or HFD supplemented with THC (100 mg/kg/day orally) for 12 weeks. We measured their body weights during the experimental period. After 12-week treatments, we performed western blotting and real-time polymerase chain reaction analyses on skin samples to evaluate the expression of inflammatory cytokines, oxidative stress markers, and autophagy markers. We observed higher tumor necrosis factor-*α* (TNF-*α*), NADPH oxidase 2 (Nox2), Nox4, and phosphorylated p65 levels; lower nuclear factor erythroid 2-related factor 2 (Nrf2) expression; and higher light chain 3 (LC3), autophagy-related 5 (Atg5), and Beclin 1 expression in the skin of HFD mice compared to the corresponding levels in the skin of mice fed with regular diet. THC administration decreased TNF-*α*, Nox2, Nox4, and phosphorylated p65 levels and activated the Nrf2 pathway. Interestingly, THC administration suppressed the expression of the autophagy markers LC3, Atg5, and Beclin 1. Overall, HFD-fed mice exhibited an elevation in inflammation, oxidative stress, and autophagy in their skin. THC ameliorated obesity-related skin pathology, and therefore, it is a potential therapeutic agent for obesity-related inflammatory skin diseases.

## 1. Introduction

Obesity, a global health problem, is a major cause of systemic metabolic inflammation and various diseases, including type 2 diabetes, dyslipidemia, hypertension, and cardiovascular diseases [1]. Obesity has also been linked to various skin conditions, such as lymphedema, acanthosis nigricans, striae distensae, and psoriasis [2–4]. Obese individuals often experience reduced skin barrier function, dry skin, and itching [5]. However, the mechanisms underlying obesity-associated skin pathologies remain unclear.

Autophagy is the primary process involved in the cellular elimination of toxic protein aggregates, damaged organelles, and invading microorganisms [6]. By degrading signaling components, autophagy can regulate the NF-*κ*B signaling pathway and affect immune responses [7]. Autophagy plays an important protective function against obesity and obesity-induced lipotoxic, proteotoxic, and oxidative stresses and thereby preserves physiological homeostasis in the human body [8]. However, obesity and its associated stresses can disrupt the autophagic process, further exacerbating obesity-related abnormalities in multiple metabolic organs [9, 10]. Autophagy is also implicated in multiple skin disorders such as infectious skin disease, psoriasis, and skin cancer [11–14]. However, to the best of our knowledge, there has been no study on the effects of obesity-induced skin pathologies on autophagy.

Curcumin (1,7-bis[4-hydroxy-3-methoxyphenyl]-1,6-heptadiene-3,5-dione) is an active component in turmeric rhizomes (*Curcuma longa* Linn.), a common spice in dishes such as curries. Curcumin has antioxidative, anti-inflammatory, antihyperlipidemic, hypoglycemic, and anticarcinogenic properties [15]. It also has protective effects against diabetes mellitus-related nephropathy, retinopathy, and vascular diseases [16]. Tetrahydrocurcumin (THC) is a reduced curcumin analog found in the gastrointestinal tract; it has a stronger antioxidant activity than curcumin [17].

Here, we aimed to determine the molecular events driving diet-induced obesity-related inflammation, oxidative stress, and autophagy in the skin. Furthermore, we examined the effects of THC on skin, including autophagy, in a diet-induced obese mouse model.

## 2. Materials and Methods

### 2.1. Animal Experiments

**Figure 1:**
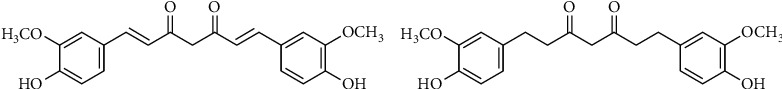
Chemical structures of curcumin and tetrahydrocurcumin.

All animal procedures were approved by the Institutional Animal Care and Use Committees of Yonsei University, Wonju, Korea (YWC-170802-2). Six-week-old male C57BL/6J mice (15 g) were purchased from Dae Han Link (Chungbuk, Korea). The mice were housed in animal rooms at a constant temperature of 20°C ± 5°C and a 12 h light/dark cycle. After a 2-week acclimatization period, the 8-week-old C57BL/6J mice were assigned to the following three groups: regular diet (*n* = 5), high-fat diet (HFD, *n* = 5), and HFD orally supplemented with 100 mg/kg/day THC (Sigma-Aldrich, St. Louis, MO, USA (96.5% purity, Figure 1)) (HFD+T100, *n* = 5). HFD provided 60% of energy in the form of fat (D12492; Research Diets, New Brunswick, NJ). 94% of fatty acids in the HFD was composed of palmitic acid (20%), stearic acid (11%), oleic acid (34%), and linoleic acid (29%). THC was provided in the mouse chow. During the 12-week experimental period, we monitored the changes in food intake and body weight of the mice on a weekly basis. At the end of the experiment, we euthanized the mice and immediately excised the shaved skin.

### 2.2. Quantitative Reverse Transcription-Polymerase Chain Reaction (qRT-PCR)

Total RNA was extracted using the RNeasy® Plus Mini Kit (Qiagen, Hilden, Germany). We used the Transcriptor First-Strand cDNA Synthesis Kit (Roche Applied Science, Mannheim, Germany) to synthesize cDNA from the total RNA (1 *μ*g). We performed qPCR in triplicate using the TaqMan™ Master Mix and Real-Time PCR system (Applied Biosystems, Foster City, CA, USA). We used the primers of *LC3* (also known as *Map1lc3a*; TaqMan Assay ID Mm00458724_m1), *Atg5* (Mm001187303_m1), *Becn1* (Mm01265461_m1), *p65* (also known as *Rela*; Mm01310735_m1), *Nox2* (Mm01287743_m1), *Nox4* (Mm00479246_m1), *Nrf2* (Mm00477784_m1), and *TNF-α* (Mm00443258_m1) in this study. We normalized the mRNA levels of *LC3*, *Atg5*, *Becn1*, *p65*, *Nox2*, *Nox4*, *Nrf2*, and *TNF-α* to that of *GAPDH* (Mm02758991_m1). Relative quantification was performed using a LightCycler® 96 Instrument (Roche Diagnostics, Mannheim, Germany).

### 2.3. Western Blotting

The mouse skin tissues were extracted in PRO-PREP™ lysis buffer (Intron, Seoul, Korea) containing protease inhibitor cocktail (Roche Diagnostics, Mannheim, Germany). We used a solution of copper (II) sulfate and bicinchoninic acid (Sigma-Aldrich, St. Louis, MO, USA) to measure the protein concentrations of the extracts. Equal amounts of protein (20 *μ*g) were separated by 10% sodium dodecyl sulfate-polyacrylamide gel electrophoresis, transferred to enhanced chemiluminescence nitrocellulose membranes (GE Healthcare, Little Chalfont, UK), and then blocked for 1 h with 5% skim milk in Tris-buffered saline containing 0.1% TWEEN® 20. The membranes were incubated overnight at 4°C with rabbit anti-LC3 (1 : 1,000; Abcam, Cambridge, MA, USA), rabbit anti-Beclin 1 (1 : 1,000; Novus Biologicals, Centennial, CO, USA), rabbit anti-Atg5 (1 : 1,000; Abcam), rabbit anti-p65 (1 : 1,000; Abcam), rabbit anti-phospho p65 (1 : 1,000; Abcam), rabbit anti-TNF-*α* (1 : 1,000; Abcam), rabbit anti-Nox2 (1 : 1,000; Abcam), rabbit anti-Nox4 (1 : 1,000; Abcam), or rabbit anti-Nrf2 (1 : 1,000; Abcam) antibodies. The primary antibodies were detected using horseradish peroxidase-conjugated goat anti-rabbit or goat anti-mouse (1 : 1,000, Abcam) IgG secondary antibodies. We visualized the protein bands using LuminoGraph II (Atto, Tokyo, Japan). Densitometric analysis was performed using ImageJ version 1.8.0 (National Institutes of Health).

### 2.4. Statistical Analyses

We conducted a one-way ANOVA with Tukey's post hoc tests to analyze the differences in body weight; blood glucose levels; TNF-*α*, Nox2, Nox4, Nrf2, LC3, Beclin 1, and Atg5 mRNA and protein expression; *p65* mRNA expression; and phosphorylated p65 protein levels among the groups. Statistical analyses were performed using GraphPad Prism version 5.01 (GraphPad Software, San Diego, CA, USA). Significance was set at *P* < 0.05.

## 3. Results

### 3.1. Tetrahydrocurcumin Treatment Lowered TNF-*α* mRNA and Protein Expressions in the Skin of Obese Mice

**Figure 2:**
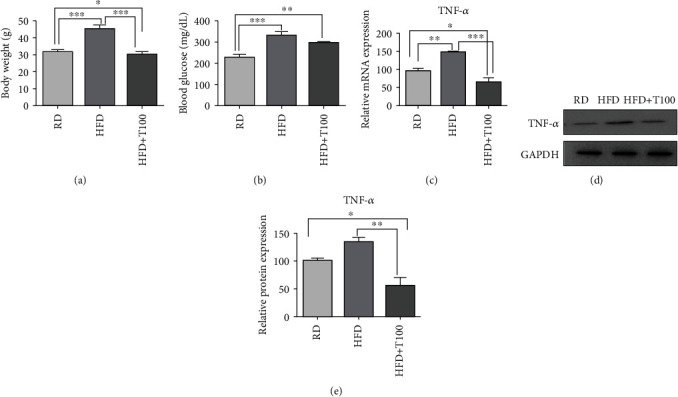
Body weight, blood glucose levels, and TNF-*α* mRNA and protein expressions in the regular diet (RD), high-fat diet (HFD), and HFD plus tetrahydrocurcumin supplementation (HFD+T100) groups. (a) After 12 weeks of treatment, the HFD group presented a significantly higher body weight than the RD group, whereas the HFD+T100 group presented a significantly lower body weight than the HFD group. (b) After 12 weeks of treatment, the HFD group presented a significantly higher blood glucose level than the RD group. The HFD+T100 group presented a lower blood glucose level than the HFD group, although the difference was not significant. (c) qRT-PCR analysis of *TNF-α* expression in the skin of RD (*n* = 5), HFD (*n* = 5), and HFD+T100 (*n* = 5) mice. Relative mRNA expression was normalized to *GAPDH* expression. Fold differences in expression were calculated using the comparative CT method, standardized to the RD value. (d) Western blotting of TNF-*α* in the skin of RD, HFD, and HFD+T100 mice. Data were normalized to GAPDH expression. Data are representative of three independent experiments. (e) Differences in western blot quantification were determined. Data are presented as mean ± standard deviation of three independent experiments. All comparisons utilized one-way ANOVA with Tukey's post hoc tests. ^∗^*P* < 0.05, ^∗∗^*P* < 0.01, and ^∗∗∗^*P* < 0.001. RD: regular diet; HFD: high-fat diet; HFD+T100: high-fat diet+tetrahydrocurcumin (100 mg/kg).

As expected, after 12 weeks of treatment, the HFD group mice presented a significantly higher body weight and blood glucose level than the regular diet group mice. The HFD+T100 group presented a significantly lower body weight than the HFD-alone group mice (Figures 2(a) and 2(b)). Furthermore, *TNF-α* mRNA expression was significantly higher in the HFD group mice than in the regular diet group mice. However, the HFD+T100 group mice had significantly lower skin *TNF-α* mRNA expression than the HFD group mice (Figure 2(c)). TNF-*α* protein expression exhibited the same patterns as mRNA expression (Figures 2(d) and 2(e)).

### 3.2. THC Alleviated Oxidative Stress in the Skin of Obese Mice

**Figure 3:**
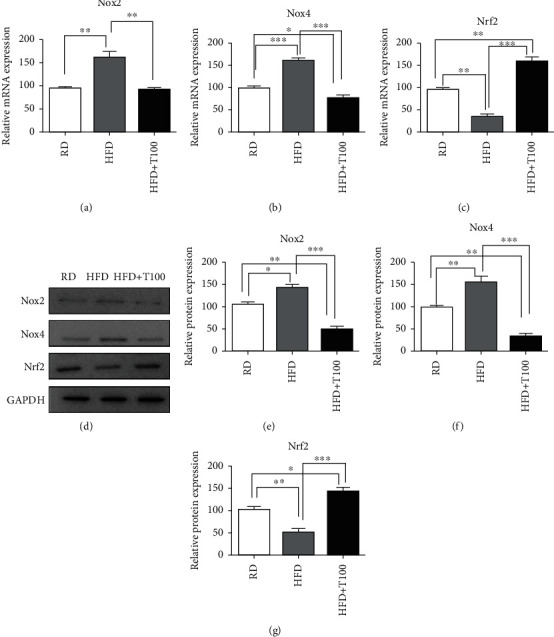
Nox2, Nox4, and Nrf2 expressions in RD, HFD, and HFD+T100 mouse skin. (a–c) qRT-PCR analysis of (a) *Nox2*, (b) *Nox4*, and (c) *Nrf2* mRNA expressions in the skin of RD (*n* = 5), HFD (*n* = 5), and HFD+T100 (*n* = 5) mice. Relative mRNA expression was normalized to *GAPDH* expression. Fold differences in the expression were calculated using the comparative CT method, standardized against the RD value. (d) Western blotting analysis of Nox2, Nox4, and Nrf2 protein expressions in the skin of RD (*n* = 5), HFD (*n* = 5), and HFD+T100 (*n* = 5) mice. Data were normalized to GAPDH expression. The results are representative of three independent experiments. (e–g) Differences in western blot quantification were determined. Data are presented as mean ± standard deviation of three independent experiments. All comparisons utilized one-way ANOVA and Tukey's post hoc tests. ^∗^*P* < 0.05, ^∗∗^*P* < 0.01, and ^∗∗∗^*P* < 0.001. RD: regular diet; HFD: high-fat diet; HFD+T100: high-fat diet+tetrahydrocurcumin (100 mg/kg).

Nox2 and Nox4 contribute to reactive oxygen species production [11], whereas Nrf2 is a component of cellular antioxidant pathways [18]. We measured Nox2, Nox4, and Nrf2 expressions to evaluate the changes in oxidative stress in the skin. The results of qRT-PCR showed that *Nox2* and *Nox4* mRNA levels were significantly higher, and the *Nrf2* mRNA level was significantly lower in the skin of HFD-fed mice than in the skin of regular diet-fed mice (Figures 3(a)–3(c)). THC treatment attenuated the HFD-induced increase in the *Nox2* and *Nox4* mRNA expressions (Figures 3(a) and 3(b)). In contrast, *Nrf2* mRNA expression in the HFD+T100 mouse skin was higher than that in the HFD mouse skin (Figure 3(c)). The expression profiles of Nox2, Nox4, and Nrf2 proteins were similar to those of the mRNA (Figures 3(d)–3(g)).

### 3.3. THC Suppressed the Induction of Autophagic Flux in the Skin of Obese Mice

**Figure 4:**
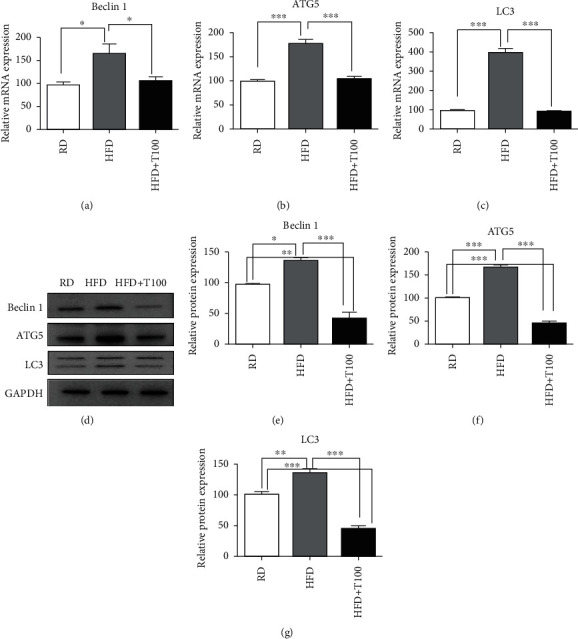
Beclin 1, Atg5, and LC3 expressions in RD, HFD, and HFD+T100 mouse skin. (a–c) qRT-PCR analysis of (a) *BECN1*, (b) *Atg5*, and (c) *LC3* expressions in the skin of RD (*n* = 5), HFD (*n* = 5), and HFD+T100 (*n* = 5) mice. Relative mRNA expression, determined by qRT-PCR, was normalized to *GAPDH* expression. Fold differences in the expression were calculated using the comparative CT method and standardizing against the RD value. (d–g) Western blotting analysis of the skin from RD (*n* = 5), HFD (*n* = 5), and HFD+T100 (*n* = 5) mice for Beclin 1, Atg5, and LC3 protein expressions. Data were normalized to GAPDH expression. The results are representative of three independent experiments. (e–g) Differences in western blot quantification were determined. Data are presented as mean ± standard deviation of three independent experiments. All comparisons utilized one-way ANOVA and Tukey's post hoc tests. ^∗^*P* < 0.05, ^∗∗^*P* < 0.01, and ^∗∗∗^*P* < 0.001. RD: regular diet; HFD: high-fat diet; HFD+T100: high-fat diet+tetrahydrocurcumin (100 mg/kg).

To investigate the effects of obesity and THC on autophagy, we measured the expression of the autophagy-related factors LC3, Beclin 1, and Atg5. The mRNA expression of *LC3*, *BECN1*, and *Atg5* in the skin of HFD-fed mice was significantly higher than that in the skin of regular diet-fed mice. However, THC treatment significantly inhibited the HFD-induced increases in *LC3*, *BECN1*, and *Atg5* mRNA expressions (Figures 4(a)–4(c)). The protein expression pattern was similar to the mRNA expression pattern in all three groups (Figures 4(d)–4(g)).

### 3.4. NF-*κ*B/p65 Signaling Pathway Mediates the Anti-Inflammatory and Antioxidant Effects of THC in the Skin of Obese Mice

**Figure 5:**
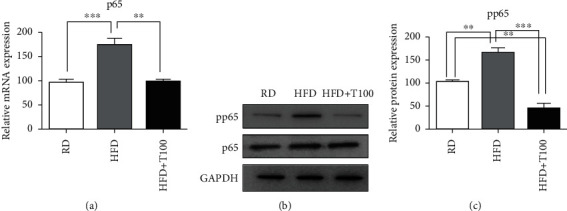
p65 expression and phosphorylation in RD, HFD, and HFD+T100 mouse skin. (a) qRT-PCR analysis of *p65* expression in RD (*n* = 5), HFD (*n* = 5), and HFD+T100 (*n* = 5) mouse skin. Relative mRNA expression, determined by qRT-PCR, was normalized to *GAPDH* expression. Fold differences in expression were calculated using the comparative CT method, standardized against the RD value. (b) Western blotting of phosphorylated p65 levels in the skin of RD (*n* = 5), HFD (*n* = 5), and HFD+T100 (*n* = 5) mice. Data were normalized to p65 and GAPDH expressions. The results are representative of three independent experiments. (c) Differences in western blot quantification were determined. Data are presented as mean ± standard deviation of three independent experiments. All comparisons utilized one-way ANOVA and Tukey's post hoc tests. ^∗∗^*P* < 0.01 and ^∗∗∗^*P* < 0.001. RD: regular diet; HFD: high-fat diet; HFD+T100: high-fat diet+tetrahydrocurcumin (100 mg/kg); pp65: phosphorylated p65.

The NF-*κ*B/p65 signaling pathway plays a central role in innate and adaptive immune responses by inducing proinflammatory genes and regulating immune cells [19]. To determine the detailed mechanisms underlying the anti-inflammatory and antioxidant effects of THC, we evaluated its effect on the NF-*κ*B/p65 expression. *p65* mRNA expression and phosphorylated p65 protein levels in the skin of HFD-fed mice were significantly higher than those in the skin of regular-diet-fed mice. *p65* mRNA expression and phosphorylated p65 protein levels were significantly lower in the skin of HFD+T100-fed mice than in the skin of HFD-fed mice (Figure 5).

## 4. Discussion

Our results supported that HFD-induced obesity affected inflammatory cytokines, oxidative stress, and autophagy markers in the skin. In terms of potential mechanisms, a recent in vivo study using an imiquimod-induced psoriatic dermatitis animal model showed that HFD exacerbates psoriatic dermatitis by increasing IL-17A-producing T cells in the skin [20]. Another study found that the typical high-fat, high-sugar western diet enhanced susceptibility to imiquimod-induced psoriatic dermatitis in mice [21]. Our research was consistent with these previous results and suggests that HFD induces skin inflammation with molecular changes in cytokine production and autophagy, although no obvious changes to skin morphology occurred.

The role of oxidative stress has been emphasized in obese patients with chronic inflammatory skin disease such as psoriasis [22]. The nuclear factor- (NF-) *κ*B pathway is related to inflammatory responses through the regulation of the innate and adaptive arms of the immune system [23]. NF-*κ*B, a redox-sensitive factor, is also associated with oxidative stress [24]. Notably, a vicious cycle has been reported between the NF-*κ*B pathway and oxidative stress. Oxidative stress is important in the activation of the NF-*κ*B pathway and inflammatory cytokines are induced following activation of this pathway, exacerbating the oxidative stress [25]. Of note, Carlsen et al. reported that NF-*κ*B is activated in the whole body in obese mice fed a HFD [26]. Furthermore, obesity makes the skin more susceptible to UVB-induced oxidative stress and activation of the NF-*κ*B signaling pathway in mice [27]. The present study showed that obesity induced the NF-*κ*B activation as well as oxidative stress in the skin.

Previous studies using both human and mouse tissues demonstrated that obesity leads to the accumulation of autophagosomes in the liver and adipose tissues [28, 29]. Notably, we found in the present study that obesity induced skin autophagy. Autophagy may be a defense mechanism component to maintain cellular homeostasis under obesity-associated stress, compensating for obesity-associated endoplasmic reticulum stress [10]. Lipotoxic activation of protein kinase C induces autophagic flux in fibroblasts, protecting the cells from apoptosis [30]. Autophagy is closely associated with inflammatory skin diseases such as psoriasis [31]. Several studies provide a glimpse of possible mechanisms. For instance, autophagy inhibition increases cellular cholesterol levels during the IL-17A-mediated inflammatory response in keratinocytes [32]. In humans and mice, keratinocyte autophagy is positively correlated with psoriasis severity [31]. Moreover, impaired keratinocyte autophagy leads to psoriatic skin inflammation by activating aryl hydrocarbon receptor signaling [12].

Curcumin suppresses HFD-induced insulin resistance and obesity by decreasing liver lipogenesis and inhibiting adipocyte inflammatory pathways [33]. Additionally, curcumin ameliorates oxidative stress and Nrf2 signaling dysfunction in the muscles of HFD-fed mice [34]. However, curcumin has low bioavailability and poor absorption, limiting its therapeutic potential [35]. One way to overcome these limitations is to use THC, which has stronger antioxidant activity [17]. Recent studies have investigated the anti-inflammatory and antioxidant roles of THC, a major active metabolite of curcumin, against tumors and inflammatory diseases [36, 37]. The compound can protect against cisplatin-induced or FK506-related nephrotoxicity by regulating inflammation and apoptosis [38]. Here, we have reported the antioxidant and anti-inflammatory effects of THC on obesity-induced skin impairment. Interestingly, these effects were associated with modulation of skin autophagy.

This study had some limitations that should be addressed in future research. First, the relationship between obesity and inflammatory skin diseases should be confirmed with further studies using animal skin disease models (e.g., imiquimod-induced psoriasiform inflammation). Second, the clinical relevance of these obesity-related effects on human skin must be verified in human research. Third, we should clarify the actual anti-inflammatory mechanism of THC in the skin, specifically determining the main target cells via in vitro experiments.

## 5. Conclusions

In summary, we examined the effects of diet-induced obesity on skin inflammation and investigated the underlying mechanisms. We found that obesity-induced skin inflammation is associated with cutaneous oxidative stress, autophagy, and the NF-*κ*B/p65 signaling pathway. Promisingly, THC ameliorated these effects. Thus, obesity management may be an effective method for promoting the recovery from skin inflammation, the latter of which can be addressed specifically through THC, a promising therapeutic. Using HFD skin disease models, future studies are needed to clarify whether there are common pathomechanisms between obesity and skin inflammation. Moreover, clinical trials are required to investigate the efficacy of THC for patients suffering not only from inflammatory skin diseases but also from obesity.

## Data Availability

The datasets analyzed during the current study are available from the corresponding author on reasonable request.
